# Associations Between Physical Activity, Muscle Mass, and Functional Outcomes in Community-Dwelling Older Adults from Chile: A Cross-Sectional Study

**DOI:** 10.3390/sports14060245

**Published:** 2026-06-16

**Authors:** Catalina Muñoz-Strale, Josivaldo De Souza-Lima, Rodrigo Yáñez-Sepúlveda, Javiera Alarcon-Aguilar, Maribel Parra-Saldias, Daniel Duclos-Bastias, Andrés Godoy-Cumillaf, Eugenio Merellano-Navarro, José Bruneau-Chávez, Claudio Farias-Valenzuela, Frano Giakoni-Ramírez

**Affiliations:** 1 Facultad de Educación y Humanidades, Escuela de Ciencias del Deporte, Universidad Andres Bello, Las Condes, Santiago 7550000, Chile; josivaldo.desouza@unab.cl (J.D.S.-L.); rodrigo.yanez.s@unab.cl (R.Y.-S.); javiera.alarcon@unab.cl (J.A.-A.); frano.giakoni@unab.cl (F.G.-R.); 2 School of Medicine, Universidad Espíritu Santo, Samborondón 092301, Ecuador; 3 Departamento de Educación Física, Deporte y Recreación, Universidad de Atacama, Copiapó 1530000, Chile; maribel.parra@uda.cl; 4 iGEO, Escuela de Educación Física, Pontificia Universidad Católica de Valparaiso, Valparaiso 2340021, Chile; dduclbas@uax.es; 5 METIS Research Lab, Facultad de Negocios y Tecnología, Universidad Alfonso X el Sabio (UAX), 28691 Madrid, Spain; 6 Grupo de Investigación en Educación Física, Salud y Calidad de Vida (EFISAL), Facultad de Educación, Universidad Autónoma de Chile, Temuco 4780000, Chile; andres.godoy@uautonoma.cl; 7 Department of Physical Activity Sciences, Faculty of Education Sciences, Universidad Católica del Maule, Talca 3530000, Chile; emerellano@ucm.cl; 8 Departamento de Educación Física, Deportes y Recreación, Universidad de la Frontera, Temuco 4811230, Chile; jose.bruneau@ufrontera.cl; 9 Escuela de Ciencias de la Actividad Física, el Deporte y la Salud, Universidad de Santiago de Chile (USACH), Santiago 9170124, Chile; claudio.farias.v@usach.cl

**Keywords:** physical activity, physical fitness, older adults, muscle mass, functional capacity

## Abstract

Population aging increases the need to preserve functional independence in older adults. This cross-sectional study examined the associations between physical activity (PA), percentage muscle mass (%Muscle), and functional outcomes in 129 community-dwelling older adults (mean age 72.05 ± 8.46 years). PA was assessed with the IPAQ (MET-min/week), body composition via bioelectrical impedance, and outcomes included the Timed Up and Go test (TUG), handgrip strength, calcaneal bone status (QUS-derived T-score), and SF-36 Physical Component Summary (PCS). Total PA showed a small positive association with handgrip strength (r = 0.19, *p* = 0.031). Bootstrapped mediation analyses (5000 resamples), adjusted for age and BMI, revealed no statistically significant indirect effects through %Muscle (all 95% CIs included zero). Exploratory simulations based on observed associations suggested modest changes in handgrip strength with hypothetical increases in PA (+0.25 kg at +20% METs; +0.62 kg at +50% METs), while changes in other outcomes were minimal. These findings indicate that, in this relatively well-functioning sample, total PA volume has limited explanatory value for variability in functional and musculoskeletal outcomes. Muscle mass did not statistically account for the PA–function relationship. Given the cross-sectional design, causal inferences cannot be drawn. These findings suggest that targeted exercise programs emphasizing muscle strength and neuromuscular function may be more effective than increases in overall physical activity volume for preserving functional capacity in older adults.

## 1. Introduction

The global population is aging rapidly, with the proportion of individuals aged 60 years and older expected to double by 2050. This demographic shift poses significant challenges to healthcare systems, particularly in maintaining functional independence and quality of life among older adults [1,2]. In Chile, the aging index has increased dramatically, reaching 79 older adults per 100 children under 15 in 2024, highlighting the urgency of addressing age-related functional decline in community settings [3]. Sarcopenia, characterized by progressive loss of muscle mass and strength, is a key contributor to frailty, falls, and loss of autonomy in this population [4,5]. Understanding modifiable factors that can mitigate these processes is therefore a public health priority.

Physical activity (PA) is one of the most effective non-pharmacological strategies to counteract age-related declines in muscle mass and function. Regular PA, particularly moderate-to-vigorous intensity activities, has been consistently associated with better physical performance, higher muscle strength, and improved health-related quality of life in older adults [6,7]. Recent meta-analyses confirm that higher levels of PA are linked to lower risk of sarcopenia and better mobility outcomes, including faster gait speed and reduced Timed Up and Go times [8,9]. However, the underlying mechanisms through which PA exerts these benefits are not fully elucidated, with growing evidence suggesting that changes in body composition, especially muscle mass, may play a central mediating role.

Skeletal muscle mass serves as a critical physiological reserve that supports mobility, metabolic health, and overall functional capacity during aging. Several cross-sectional and longitudinal studies have demonstrated that muscle mass partially mediates the relationship between physical activity and functional outcomes such as balance, strength, and daily living activities [10,11]. For instance, higher muscle mass has been shown to explain part of the protective effect of PA against falls and frailty in community-dwelling older populations [12,13]. Despite these findings, few studies have formally tested the mediating role of muscle mass in Latin American samples, where cultural, dietary, and socioeconomic factors may influence these associations.

In Chile, physical inactivity remains highly prevalent among older adults, contributing to accelerated sarcopenia and reduced quality of life [14,15]. Community-based data indicate that only a small proportion of Chilean older adults meet recommended physical activity levels, with women being particularly affected. This context underscores the need for locally relevant evidence on how physical activity influences functional outcomes and whether muscle mass acts as a key mediator in this relationship. Such information is essential for designing targeted interventions that can be implemented in community settings across the country.

The present study aimed to examine the mediating effect of percentage muscle mass (%Muscle) on the association between total physical activity (measured in MET-min/week) and key functional outcomes, including mobility (TUG), handgrip strength, calcaneal bone status (QUS-derived T-score), and the physical component of quality of life (SF-36 PCS) in community-dwelling older adults from the Metropolitan Region of Chile. We hypothesized that %Muscle would partially mediate these relationships, with higher physical activity levels associated with greater muscle mass and, in turn, better functional performance.

It should be noted, however, that contemporary literature increasingly challenges the primacy of muscle mass as the central explanatory construct for functional outcomes in older adults. Emerging evidence suggests that muscle strength, power, and quality, rather than muscle quantity per se, are more proximal determinants of physical performance, mobility, and independence [16,17,18]. Muscle quality, defined as the force produced per unit of muscle mass, integrates neuromuscular, architectural, and metabolic properties that are not captured by mass-based measures such as BIA-derived %Muscle. Similarly, muscle power has shown stronger associations with functional capacity than either muscle mass or maximal strength in older populations [17]. In this context, the present study’s use of %Muscle as a mediator represents a pragmatic choice based on the accessibility of BIA in community settings, rather than a theoretical claim that mass is the most relevant muscular construct. The findings should therefore be interpreted within this conceptual limitation, and the absence of mediation through %Muscle does not preclude the possibility that functional pathways mediated by muscle strength or quality may exist.

To enhance clinical interpretability, we also conducted exploratory predictive simulations to estimate the expected changes in functional outcomes following hypothetical increases of +20% and +50% in total METs. This approach allows for a better understanding of the potential magnitude of associations between physical activity and outcomes in this sample. By integrating statistical mediation analysis with exploratory simulation, the study provides associational evidence and practical implications for community-based geriatric research.

In summary, this cross-sectional investigation contributes to the growing body of evidence on the pathways linking physical activity, muscle mass, and functional health in aging populations. The findings may inform the development of more effective, mechanism-driven interventions aimed at preserving independence and quality of life in community-dwelling older adults.

## 2. Materials and Methods

### 2.1. Study Design

This cross-sectional study adopted a quantitative, non-experimental design with both descriptive and analytical objectives. Participants were recruited through convenience sampling from community centers in the Metropolitan Region of Santiago, Chile.

### 2.2. Participants

A total of 129 community-dwelling older adults (98 women and 31 men) participated in the study. The mean age was 72.05 ± 8.46 years (range 60–91 years), and the mean body mass index (BMI) was 27.8 ± 3.6 kg/m^2^.

Inclusion criteria were: (1) age ≥ 60 years, (2) independent community living, (3) ability to perform basic activities of daily living without assistance, and (4) provision of written informed consent.

Exclusion criteria included acute medical conditions or physical limitations that would prevent safe completion of the functional assessments.

No a priori sample size calculation was conducted given the exploratory nature of the study. A post hoc sensitivity analysis [19] indicated that with n = 129, α = 0.05, and a small indirect effect (standardized a × b ≈ 0.14), the estimated post hoc power was 0.72–0.78, suggesting adequate power to detect small-to-moderate but not very small indirect effects.

### 2.3. Measurements

All assessments were performed in a controlled indoor environment by trained professionals following standardized protocols suitable for older adults.

### 2.4. Anthropometric and Body Composition Assessment

Body mass index (BMI), percentage body fat (%Fat), and percentage muscle mass (%Muscle) were estimated using bioelectrical impedance analysis (BIA; Omron HBF-514C, Omron Healthcare, Kyoto, Japan). All measurements were taken in the morning, with participants wearing light and comfortable clothing, in a standing position with legs 35–45° apart and arms extended at a 90° angle perpendicular to the body without bending the elbows, following a minimum 3-h fast and without having exercised in the previous 3 h. For each measurement, the device was disconnected and reconnected, and all required variables were re-entered prior to assessment, following the validation protocol described by Moreno et al. (2001) [20]. Body height was measured in a bipedal position using a Seca 213 stadiometer (Seca GmbH, Hamburg, Germany), and BMI was calculated as body weight divided by height squared (kg/m^2^). Body circumferences (waist, arm, and thigh) were measured using a flexible anthropometric tape following the standardized protocols of the International Society for the Advancement of Kinanthropometry (ISAK). All measurements were taken at the anatomical landmarks defined by ISAK: waist circumference at the midpoint between the lower costal border and the iliac crest; mid-upper arm circumference at the midpoint between the acromion and olecranon processes; and mid-thigh circumference at the midpoint between the greater trochanter and the lateral tibial condyle. Each measurement was taken twice by the same trained evaluator (ISAK Level 1 certified); in cases where the two values differed by more than 1%, a third measurement was taken, and the median of the three values was used. To minimize measurement bias, all assessments were performed by the same evaluator throughout the data collection period, following a standardized measurement order and using a single calibrated instrument.

### 2.5. Calcaneal Bone Status

Calcaneal bone status was assessed using a quantitative ultrasound device (QUS; OsteoSys Sonost-3000, OsteoSys Co., Seoul, South Korea). Measurements were performed at the calcaneus, with participants placing their bare foot on the device platform according to the manufacturer’s standardized protocol. Device calibration was verified prior to data collection using the manufacturer’s internal phantom (S/N: P1503018S, VER 0.2), with reference values of SOS: 1638 m/s and BUA: 23 dB/MHz, both within the manufacturer’s valid ranges (SOS: 1622–1651 m/s; BUA: 15–30 dB/MHz at 15–30 °C). For descriptive purposes results are expressed as T-scores according to World Health Organization (WHO) criteria (T-score ≥ −1.0: normal; −2.5 to −1.0: osteopenia; ≤−2.5: osteoporosis).

### 2.6. Functional Performance

Functional capacity was evaluated using the following validated tests:

Timed Up and Go (TUG) test: to assess mobility and dynamic balance.

30-s Chair Stand Test: lower-limb strength.

30-s Arm Curl Test: upper-limb strength.

Handgrip strength (kg) was measured in the dominant hand using a calibrated hand dynamometer. Participants were seated with shoulders adducted, elbow flexed at 90°, and wrist in neutral position. Two maximal efforts of 3 s each were performed, with 1-min rest between trials. The highest value was recorded for analysis.

### 2.7. Cardiovascular Measures

Resting systolic and diastolic blood pressure were measured using a validated sphygmomanometer following European Society of Cardiology guidelines.

### 2.8. Physical Activity

Physical activity levels were assessed using the long version of the International Physical Activity Questionnaire (IPAQ). Data was expressed in MET-min/week. To minimize overestimation, values were truncated according to official IPAQ guidelines (maximum: 10,080 MET-min/week for vigorous, 5040 for moderate, and 15,120 for total activity).

### 2.9. Health-Related Quality of Life

Quality of Life was evaluated with the Short Form-36 Health Survey (SF-36). The Physical Component Summary (PCS) and Mental Component Summary (MCS) scores were calculated using standardized scoring algorithms.

### 2.10. Statistical Analysis

Descriptive data are presented as means ± standard deviations. Bivariate associations were examined using Pearson’s correlation coefficients, with effect sizes interpreted as trivial (<0.10), small (0.10–0.29), medium (0.30–0.49), and large (≥0.50). Given the cross-sectional design of the study, the mediation analyses conducted are statistical (associational) in nature and do not permit causal or temporal inferences regarding the directionality or mechanisms of the relationships [21,22].

Bootstrapped mediation analyses (5000 resamples) were performed using regression-based methods to examine whether percentage muscle mass (%Muscle) statistically accounted for the association between total physical activity (METs Total) and functional outcomes. Multiple linear regression models were used, adjusting for age and BMI as covariates. Given the marked sex imbalance in the sample (n = 98 women, n = 31 men), sex was not included as a covariate in the primary mediation models to avoid overfitting, as the male subgroup (n = 31) falls below the minimum recommended for reliable bootstrapped indirect effect estimation. Indirect effects (ab), bias-corrected accelerated 95% confidence intervals, and exact *p*-values are reported. Multicollinearity was assessed using variance inflation factors (VIF); all VIF values were below 3.0 (range: 1.03–2.87), indicating no problematic multicollinearity. Residual diagnostics confirmed approximate linearity and homoscedasticity (Breusch-Pagan test: *p* > 0.10 for all models). All statistical analyses were conducted in Python (version 3.11) using statsmodels and pandas packages. Statistical significance was set at *p* < 0.05.

Additionally, exploratory predictive simulations were conducted to illustrate the potential magnitude of change in outcomes associated with hypothetical increases in total physical activity. Simulations were derived from the unstandardized partial regression coefficients (β) obtained from the adjusted linear regression models (PA → outcome, controlling for age and BMI). Hypothetical increments of +20% and +50% above the observed sample mean MET-min/week were calculated after re-applying IPAQ truncation rules (maximum 180 min/day per activity domain). The estimated change in each outcome was computed as: Δoutcome = βMETs × ΔMETs, where βMETs represents the partial regression coefficient for METs Total from the adjusted model and ΔMETs represents the hypothetical increment in total PA. All simulated values are purely exploratory and hypothetical; they are based exclusively on cross-sectional associations observed in this sample and must not be interpreted as causal effects, longitudinal trajectories, or expected outcomes of any intervention. Python (version 3.11) code used to generate these estimates is available from the corresponding author upon reasonable request.

### 2.11. Ethical Considerations

The study was conducted in accordance with the Declaration of Helsinki (2024) [23]. and national regulations governing research with human participants. All participants received detailed information about the study objectives and procedures and provided written informed consent. Participation was voluntary, and individuals could withdraw at any time without consequences. Data was anonymized using numerical codes to ensure confidentiality. The study received ethical approval from the Ethics Committee of Universidad Autónoma de Chile (CEC-2320, 2020).

## 3. Results

### 3.1. Descriptive Characteristics of the Participants

The sample consisted of older adults with a mean age of 72.1 ± 8.5 years and an average BMI within the overweight range. Body composition analysis showed relatively high adiposity (%Fat: 39.3 ± 7.6) alongside moderate muscle mass (%Muscle: 24.3 ± 4.8), consistent with age-related physiological changes. Physical activity levels, expressed as MET-min/week, exhibited substantial variability across participants, even after applying IPAQ truncation procedures, indicating heterogeneous behavioral patterns within the sample.

From a functional perspective, the mean Timed Up and Go (TUG) time (9.10 ± 3.34 s) suggests generally preserved mobility, although the upper range indicates the presence of individuals with functional limitations. Handgrip strength values were moderate but widely distributed, reflecting variability in muscular capacity. Bone health indicators (T-score: −2.38 ± 0.97) suggest that a proportion of participants may present reduced bone density. Cardiovascular measures were within expected ranges, while SF-36 PCS scores indicate moderate perceived physical health.

Bivariate analysis revealed limited associations between total physical activity and the selected outcomes. A small but statistically significant positive correlation was observed between METs Total and handgrip strength (r = 0.19, *p* = 0.031). No significant associations were found for the remaining variables, including body composition, functional performance, bone density, and quality of life. Overall, the magnitude of correlations was trivial to small, suggesting that total physical activity alone does not strongly explain variability in these outcomes.

Mediation analysis did not support a significant indirect effect of physical activity through percentage muscle mass. All indirect effects were small and non-significant, with confidence intervals crossing zero in every model. These findings indicate that %Muscle does not act as a meaningful mediator between physical activity and the evaluated outcomes after adjusting for age and BMI.

Simulation analyses showed that increases of +20% and +50% in physical activity were associated with modest absolute changes in the outcomes. The most consistent effect was observed in handgrip strength, with increases of 0.25 and 0.62 kg, respectively. Other variables showed minimal changes, including slight reductions in TUG time and %Fat, small increases in SF-36 PCS, and negligible variation in T-score. These results suggest that, although increased physical activity is associated with favorable trends, the magnitude of change remains limited at the individual level.

The descriptive characteristics of the sample are presented in Table 1, highlighting a predominantly older population with overweight status and considerable variability in physical activity levels.

Bivariate associations between total physical activity and the selected outcomes are shown in Table 2, with only handgrip strength demonstrating a statistically significant relationship.

**Figure 1:**
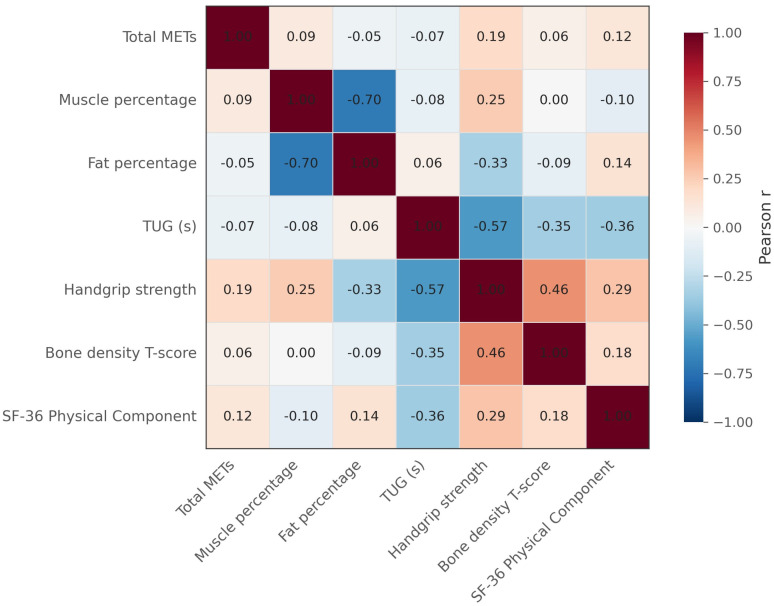
Pearson Correlation Heatmap between Physical Activity, Body Composition, and Functional Outcomes in Older Adults.

The correlation structure among physical activity, body composition, and functional outcomes is illustrated in Figure 1, highlighting generally weak associations between total METs and most variables, alongside stronger relationships among functional and body composition indicators.

Figure 1 Pearson correlation heatmap showing the relationships between total physical activity (METs), body composition (% muscle and % fat), and key functional outcomes (TUG test, handgrip strength, bone density T-score, and SF-36 physical component) in 129 community-dwelling older adults. Warmer colors (red) indicate positive correlations and cooler colors (blue) indicate negative correlations. Values represent Pearson’s r coefficients.

**Figure 2:**
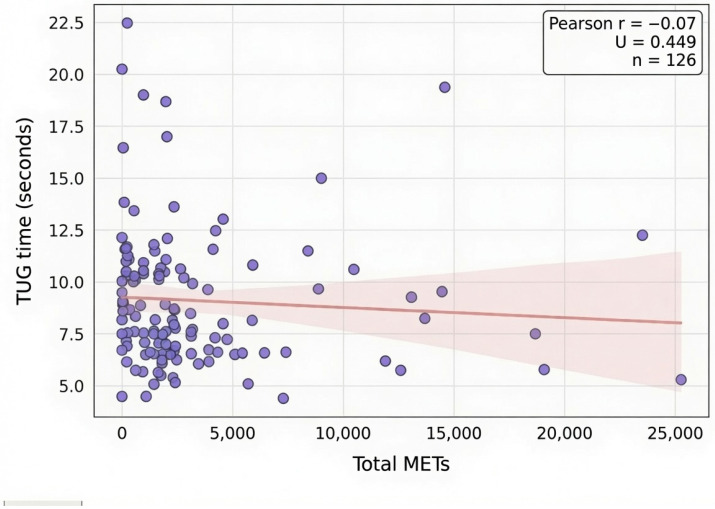
Relationship between Total Physical Activity (MET-min/week) and Timed Up and Go Test Performance in Older Adults.

The scatterplot (Figure 2) indicates a weak and non-significant inverse relationship between total physical activity and TUG performance, suggesting limited association between activity levels and mobility in this sample.

Figure 2 Scatterplot showing the association between total physical activity (METs Total) and Timed Up and Go (TUG) test time (seconds). The red line represents the linear regression fit, and the shaded area indicates the 95% confidence interval. Pearson’s correlation coefficient (r = −0.07, *p* = 0.317, n = 129) suggests a weak and non-significant negative association.

**Figure 3:**
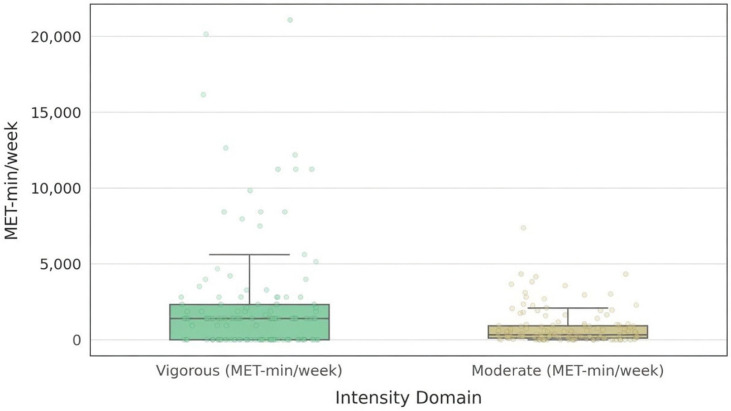
Distribution of Self-Reported Physical Activity by Intensity Domain (MET-min/week).

The distribution of physical activity by intensity domain (Figure 3) shows marked variability and right-skewness in vigorous activity, whereas moderate activity appears more concentrated at lower values.

Figure 3 Boxplot showing the distribution of self-reported physical activity by intensity domain (vigorous and moderate MET-min/week) after applying IPAQ truncation guidelines. Individual data points are shown with low transparency. Note the high inter-individual variability, particularly in the vigorous intensity domain.

The results of the mediation analysis are summarized in Table 3, indicating no significant indirect effects of physical activity through muscle mass.

### 3.2. Exploratory Predictive Simulations

To illustrate the practical magnitude of the observed (albeit weak) associations between physical activity and the outcomes, we conducted exploratory predictive simulations. These simulations estimated the expected changes in key functional outcomes associated with hypothetical increases of +20% and +50% in total MET-min/week (with IPAQ truncation rules reapplied). These simulations are purely exploratory and hypothetical. They are based solely on the cross-sectional associations observed in this sample and should not be interpreted as evidence of causal effects or as predictions of real-world intervention outcomes.

The results of these simulations are presented in Table 4.

## 4. Discussion

The main findings of this cross-sectional study indicate that total physical activity volume showed limited associations with most functional, body composition, bone health, and quality-of-life outcomes in this sample of community-dwelling older adults. The only statistically significant association observed was a small positive correlation between total MET-min/week and handgrip strength (r = 0.19, *p* = 0.031). No meaningful associations were found with %Muscle, TUG performance, calcaneal bone status (QUS-derived T-score), or SF-36 PCS. Consistent with this, bootstrapped mediation analyses showed no statistically significant indirect effects through percentage muscle mass. Furthermore, it should be acknowledged that the bone density values reported in this study were obtained via calcaneal QUS, which measures Speed of Sound (SOS) and Broadband Ultrasound Attenuation (BUA) and provides T-score estimates that are not directly comparable to those derived from axial DXA. Calcaneal QUS is a validated screening tool for population-level bone health assessment, but its sensitivity and specificity for detecting osteoporosis differ from those of DXA [24,25]. This methodological consideration may have influenced the observed associations and should be taken into account when interpreting the bone-related findings.

From a descriptive perspective, the sample presented a profile characteristic of community-dwelling older adults, with an average BMI in the overweight range and relatively high adiposity accompanied by moderate percentage muscle mass. This pattern is consistent with population-based studies in Chile and Latin America, which describe age-related shifts in body composition characterized by increased fat mass and gradual declines in muscle mass [26,27,28,29]. Physical activity levels showed substantial inter-individual variability, even after applying IPAQ truncation procedures, reflecting the heterogeneous behavioral patterns commonly observed in older populations [30].

Functionally, mean TUG performance (9.10 ± 3.34) indicates generally preserved mobility and a low average risk of falls, although the wide range suggests the presence of individuals with emerging functional limitations [31]. This relatively good baseline functional status may partially explain the weak associations observed between physical activity and functional outcomes. In samples with preserved mobility and independence, an “ceiling effect” can reduce the observable impact of physical activity on performance-based measures, even when physiological benefits are present [32,33].

The only outcome significantly associated with total physical activity was handgrip strength, a widely recognized marker of overall muscular function and health status in older adults [6,34]. This finding aligns with evidence showing that habitual physical activity is more consistently related to strength outcomes than to complex functional tests or body composition indices when activity is assessed by self-report [35,36]. Handgrip strength may therefore represent a more sensitive indicator of the functional benefits of physical activity in relatively robust community-dwelling populations.

Contrary to our initial hypothesis, mediation analyses did not support a significant role of percentage muscle mass as an intermediary between physical activity and functional or health-related outcomes. All indirect effects were small and non-significant after adjustment for age and BMI. This finding contrasts with previous studies reporting mediating roles of percentage muscle mass, muscle power, or sarcopenic status in the relationship between physical activity and functional outcomes [10,11,37]. Several factors may explain this discrepancy. First, percentage muscle mass alone may be an insufficient mediator when muscle function, quality, or power, rather than quantity, are the primary determinants of performance [5,38]. Second, the weak association observed between physical activity and muscle mass in this sample limits the potential magnitude of any indirect effect.

Additionally, physical activity was assessed using a self-report questionnaire, which is known to introduce measurement error and attenuate associations with physiological outcomes [39]. Questionnaire-based estimates of total METs may inadequately capture the type, intensity, and mechanical loading of activities required to induce meaningful adaptations in percentage muscle mass or bone density [40,41]. This may partly explain the absence of significant associations with body composition and T-score, as well as the minimal simulated changes observed even under hypothetical increases of +20% and +50% in physical activity.

The exploratory predictive simulations further illustrated that even substantial hypothetical increases in total physical activity volume (+20% and +50% METs) were associated with only modest changes in handgrip strength and minimal changes in other outcomes. The modeled absolute changes were negligible and fell well below established clinical significance thresholds: the minimal detectable change for TUG in community-dwelling older adults [42], the minimal clinically important difference range for handgrip strength of 5.0–6.5 kg [43], and the MCID for the SF-36 PCS of approximately 3 points [44]. Importantly, given the cross-sectional design, these simulations are purely illustrative and should not be interpreted as evidence of causal intervention effects [40]. This observation is consistent with evidence indicating that resistance and multicomponent exercise programs are more effective than general increases in activity volume for improving strength, function, and frailty-related outcomes [40,41,45].

From a public health perspective, these findings highlight the importance of moving beyond total physical activity volume when designing interventions for older adults [38,39]. While maintaining adequate levels of physical activity remains essential for overall health, targeted programs that explicitly address muscle strength, balance, and neuromuscular function may be required to achieve substantial functional gains, particularly in populations with preserved baseline function [38,39,41].

Several limitations should be considered when interpreting these findings. First, the cross-sectional design precludes causal inference and any conclusions regarding temporal ordering or directionality of the associations. The mediation analyses reported are statistical (associational) in nature and should not be interpreted as evidence of mechanism or pathway. Second, the use of the IPAQ long version to assess physical activity is subject to well-documented limitations in older adults, including overestimation of activity volume, social desirability bias, and recall error, as documented for both the short and long versions of the IPAQ [46,47]. The high inter-individual variability in MET-min/week (SD ≈ 3500) likely reflects a combination of genuine heterogeneity and measurement noise. Future studies should incorporate objective activity monitoring (e.g., accelerometry) to reduce these limitations. Third, the use of BIA to estimate %Muscle provides a measure of relative muscle quantity but does not capture muscle quality, strength, power, or neuromuscular function, which are increasingly recognized as more proximal determinants of functional performance in older adults. Additionally, bone status was assessed using calcaneal quantitative ultrasound (QUS) rather than dual-energy X-ray absorptiometry (DXA), the gold standard for bone mineral density measurement. QUS-derived T-scores provide useful population-level screening information but are not directly equivalent to DXA-based BMD values. Future studies should consider axial DXA to obtain more precise bone density measurements and allow direct comparison with established normative data. Fourth, the predominance of women (76%) limits generalizability to men, frail individuals, or institutionalized populations. The marked sex imbalance (76% women) precluded the inclusion of sex as a covariate in the primary mediation models to avoid overfitting given the sample size (n = 129). Fifth, the absence of structured health characterization beyond the measures directly collected in this study. No formal comorbidity assessment was conducted, such as the Charlson Comorbidity Index or equivalent, and no systematic review of participants’ medication use was performed. Both factors could have meaningfully influenced the observed associations: chronic conditions such as type 2 diabetes, cardiovascular disease, chronic obstructive pulmonary disease, or musculoskeletal disorders are known to affect muscle mass, physical activity capacity, and functional performance independently of habitual PA levels [48]. Similarly, polypharmacy, highly prevalent in community-dwelling older adults, can influence body composition, muscle function, and exercise tolerance through multiple mechanisms [49]. The absence of these data limits our ability to rule out residual confounding and to characterize the health profile of participants with sufficient precision for clinical translation. Future studies should incorporate validated comorbidity screening tools and medication review protocols as part of baseline data collection. Finally, the absence of an a priori power calculation means the study may have been underpowered for very small indirect effects; the post hoc power estimate was 0.72–0.78.

Future research should prioritize longitudinal designs and randomized community-based trials to evaluate temporal mediation pathways, incorporate objective measures of physical activity (e.g., accelerometry), and examine mediators more proximal to performance, such as muscle strength, power, and quality. Stratification by baseline functional status (robust vs. pre-frail/frail) may also help identify subgroups in which muscle-related mechanisms play a more substantial role.

## 5. Conclusions

In this cross-sectional study of community-dwelling older adults from Chile, total self-reported physical activity showed weak and largely non-significant associations with functional performance, percentage muscle mass, bone density, and physical quality of life. Statistical mediation through percentage muscle mass (%Muscle) was not supported after adjustment for age and BMI. Handgrip strength was the only outcome demonstrating a small positive association with total PA (r = 0.19). Given the cross-sectional design, convenience sampling, and the limitations of self-reported PA and BIA-derived body composition, these findings should be interpreted cautiously and do not support causal conclusions. These results suggest that targeted exercise programs emphasizing muscle strength and neuromuscular function may be more effective for preserving functional capacity in community-dwelling older adults than increases in overall PA volume alone. Longitudinal and intervention-based studies incorporating objective PA measurement are needed to confirm these patterns.

## Figures and Tables

**Table 1:** Descriptive characteristics of the sample (N = 129).

Domain	Variable	Mean ± SD	Range
Demographics	Age (years)	72.05 ± 8.46	60.00–91.00
Body composition	BMI (kg/m^2^)	27.91 ± 4.07	20.00–43.07
Body composition	Body fat (%)	39.26 ± 7.61	21.80–54.70
Body composition	Muscle mass (%)	24.30 ± 4.77	10.20–34.60
Physical activity	METs Total (MET-min/week)	2913.95 ± 3496.09	0.00–17,160.00
Physical activity	METs Vigorous	2065.12 ± 2783.29	0.00–9600.00
Physical activity	METs Moderate	848.84 ± 1209.31	0.00–7560.00
Functionality	TUG (s)	9.10 ± 3.34	4.40–22.47
Functionality	Handgrip strength (kg)	22.58 ± 8.95	4.00–45.00
Bone health	T-score	−2.38 ± 0.97	−4.10–0.20
Cardiovascular	Systolic BP (mmHg)	129.90 ± 16.86	95.00–180.00
Cardiovascular	Diastolic BP (mmHg)	78.03 ± 10.36	58.00–120.00
Quality of life	SF-36 PCS	48.39 ± 8.85	21.08–65.88

Note: Values are presented as mean ± SD and observed range. Physical activity variables were calculated following IPAQ truncation guidelines.

**Table 2:** Pearson correlations between total physical activity and outcomes.

Variable	r	*p*-Value	Magnitude
Muscle mass (%)	0.14	0.281	Small
Body fat (%)	−0.10	0.453	Trivial
TUG (s)	−0.07	0.317	Trivial
Handgrip strength (kg)	0.19	0.031 *	Small
T-score	0.07	0.455	Trivial
SF-36 PCS	0.12	0.191	Small

Note: Pearson correlations using pairwise complete cases. * *p* < 0.05.

**Table 3:** Bootstrapped mediation analysis (%Muscle as mediator).

Outcome	Indirect Effect (ab)	95% CI	*p*-Value
TUG (s)	−0.0000	−0.0001 to 0.0001	0.472
Handgrip strength (kg)	0.0001	−0.0002 to 0.0005	0.388
Body fat (%)	−0.0002	−0.0007 to 0.0003	0.312
T-score	0.0000	−0.0000 to 0.0000	0.704
SF-36 PCS	−0.0001	−0.0010 to 0.0004	0.670

Note: Models adjusted for age and BMI. 95% CI based on 5000 bootstrap samples (bias-corrected accelerated). No indirect effects were statistically significant.

**Table 4:** Exploratory simulations of hypothetical increases in physical activity.

Outcome	+20% METs	+50% METs	Interpretation
TUG (s)	−0.01	−0.02	Below MDC [19]
Handgrip strength (kg)	0.25	0.62	Below MCID range (5.0–6.5 kg) [20]
Body fat (%)	−0.12	−0.29	Negligible absolute change
SF-36 PCS	0.38	0.93	Below MCID (≈3 pts) [21]
T-score	0.00	0.01	Negligible absolute change

Note: Estimates derived from adjusted models (age and BMI). Values represent modeled absolute changes.

## Data Availability

The dataset generated and analyzed during the current study is available from the corresponding author upon reasonable request. The data are not publicly available due to ethical restrictions and participant confidentiality.

## Abbreviations

The following abbreviations are used in this manuscript:

BIA	Bioelectrical Impedance Analysis
BMI	Body Mass Index
BP	Blood Pressure
CI	Confidence Interval
IPAQ	International Physical Activity Questionnaire
ISAK	International Society for the Advancement of Kinanthropometry
METs	Metabolic Equivalents of Task
MCS	Mental Component Summary (SF-36)
PA	Physical Activity
PCS	Physical Component Summary (SF-36)
QUS	Quantitative Ultrasound
SF-36	36-Item Short Form Health Survey
TUG	Timed Up and Go test
WHO	World Health Organization
